# National Growth Charts for United Arab Emirates Children With Down Syndrome From Birth to 15 Years of Age

**DOI:** 10.2188/jea.JE20130081

**Published:** 2015-01-05

**Authors:** Elhadi H Aburawi, Nicolas Nagelkerke, Asma Deeb, Shahrban Abdulla, Yousef M. Abdulrazzaq

**Affiliations:** 1 Department of Paediatrics, College of Medicine and Health Sciences, United Arab Emirate University, Al Ain, United Arab Emirates; 2 Institute of Public Health, College of Medicine and Health Sciences, United Arab Emirate University, Al Ain, United Arab Emirates; 3 Al Mafraq Hospital, Abu Dhabi Health Authority, Abu Dhabi, United Arab Emirates; 4 Latifa Hospital, Dubai Health Authority, Dubai, United Arab Emirates

**Keywords:** growth charts, Down syndrome, height, weight, United Arab Emirates

## Abstract

**Background:**

Specific centile growth charts for children with Down syndrome (DS) have been produced in many countries and are known to differ from those of normal children. Since growth assessment depends on the growth pattern characteristic for these conditions, disorder-specific charts are desirable for various ethnic groups.

**Aims:**

To provide cross-sectional weight, height, and head circumference (HC) references for healthy United Arab Emirates (UAE) children with DS.

**Methods:**

A retrospective and cross-sectional growth study of Emirati children with DS, aged 0 to 18 years old, was conducted. Height, weight, and HC were measured in each child. Cole’s LMS statistical method was applied to estimate age-specific percentiles, and measurements were compared to UAE reference values for normal children.

**Results:**

Incidence of DS in the UAE population is 1 in 374 live births (267 in 10 000 live births). We analyzed 1263 growth examinations of 182 children with DS born between 1994 and 2012. The male-to-female ratio was 1.6:1. Height, weight, and HC centile charts were constructed for ages 0 to 13 years. The prevalence of overweight and obesity in DS children aged 10 to 13 years of age was 32% and 19%, respectively. The DS children were significantly shorter and heavier than normal children in the UAE.

**Conclusions:**

Weight, height, and HC growth charts were created for children with DS. These can be used as a reference standard for the UAE children with DS. Overweight and obesity are quite common in DS children ≥10 years of age, as DS children tend to be shorter and heavier than non-DS children.

## INTRODUCTION

The United Arab Emirates (UAE) is a mixed society, with UAE nationals (Emiratis) and nationals of 120 other countries comprising a total estimated population in 2011 of 8 264 070, of which around 1 million are Emiratis. The crude birthrate of the UAE population in 2011 was 15.76/1000 population, with a total of 83 950 births.^1^ Worldwide, overall incidence of Down syndrome (DS) is 1/600–1/800 live births. In a study done in the UAE,^2^ DS was found to occur in 1 out of 319 live births in Emiratis (31.3/10 000 live births) and 1 in 602 in non-Emiratis (16.6/10 000 live births). DS is a genetic disorder caused by the presence of a part or a full extra copy of chromosome 21 resulting in 47 chromosomes, and the most common type is meiotic non-disjunction causing trisomy 21 (95%); other types are Robertsonian translocation and mosaic type. DS children have typical dysmorphic features and cognitive impairment. They are known to be shorter than their normal counterparts and may suffer a multitude of debilitating problems, including congenital heart disease, gastrointestinal anomalies, leukemia, Alzheimer’s disease, immune dysfunction, hypothyroidism, diabetes mellitus, and vision and hearing problems.

Clinical growth percentile charts illustrate the distribution of selected body measurements in children and are used by pediatricians and other health care professionals to estimate the height-weight changes over time and to assess nutritional status among populations of children. The outer limits of “normality” are given by the 3rd and 97th percentiles of these charts. Children with certain diseases, especially chromosomal defects such as DS and Turner syndrome, follow distinct growth-curve patterns that deviate significantly from those of normal children. There have been a few publications on growth and BMI standard reference charts in normal and obese UAE children, but no publications have addressed growth charts in UAE DS patients so far.^3^^–^^5^ Since children with DS are known to have different growth patterns than normal children, it is critically important to utilize syndrome-specific growth charts when evaluating growth.

Growth charts for DS have been published for several populations; the first growth chart used specifically for DS was developed by Roche,^6^ in 1965, and a subsequent growth chart was published in 1974 by Rarick and Seefeldt, who described growth in DS children institutionalised in the United States.^7^ In the United States, Cronk et al^8^ studied 780 children with DS and established growth charts for American children with DS. The height of children with DS varies by country, which can be seen in studies that established growth charts of DS children for Holland,^9^ Sicily,^10^ Portugal,^11^ the United Kingdom,^12^ and Sweden.^13^ These studies revealed that Dutch, Swedish, English, and Portuguese DS children were taller than corresponding American children and much taller than their Sicilian counterparts.

In this study, we aimed to construct specific centile growth charts for weight, height, and head circumference (HC) that could be used as a reference standard for Emirati children with DS.

## METHODS

A national cross-sectional study combined with a retrospective longitudinal growth survey of children with DS was conducted. The study was longitudinal, as measurements were noted in each individual child from birth to current age. It was also cross-sectional, as measurements from all children were pooled at each age in order to construct the growth charts. To collect representative nationwide data, all specialized regional pediatric outpatient clinics, associations, and special centres for children with DS in 3 cities in the UAE were approached. The hospital-based clinics provide standard medical care for children with DS. The associations and special centres are run by different authorities looking after children with special needs.

At the clinics, all data were retrospectively collected by a research assistant, who is a trained nurse, from medical records in the period between September 2011 and May 2012. The diagnosis of DS had been based on the presence of typical features of DS (hypotonia, flat face, flat occiput, slanted palpebral fissures and epicanthic folds, Brushfield spots, simian crease, short broad hands, hypoplasia of middle phalanx of 5th finger, and high arched palate) and genetic tests. Additional data from the Down Syndrome Association and centres for special needs were collected by completing standard forms. All measurements were carried out in accordance with the protocol.

Children with DS were identified through the medical records of 3 Emirati districts: Al-Ain, Abu Dhabi, and Dubai. All DS patients meeting the criteria for inclusion in the study and attending the selected healthcare facilities in Dubai were selected and invited to the outpatient clinics, where their measurements were taken by a research assistant and medical students. Their medical charts were reviewed, and all previous weight, height, and HC measurements were also recorded. Any results of chromosomal studies were also recorded. In order to compute the incidence of DS in the Emirati population of Al Ain, numbers of live births of Emiratis born from April 2008 to March 2014 in one hospital were taken. This is the period for which reliable data are available. The number of Emirati live births with DS in this period was also recorded.

The study was approved by the ethics committees in these regions. Written consent was obtained from their guardians.

### Inclusion and exclusion criteria

Emirati children with DS who were born at full term, were otherwise healthy, had no diseases that could affect their growth pattern, and were aged 18 years or younger were included in the study. Children with hypothyroidism on thyroxin treatment were also included. Children who suffered from chronic diseases such as non-iron-deficiency anemia and diabetes mellitus—and those who had congenital malformations and debilitating chronic diseases like asthma or coeliac disease were excluded. Moreover, children whose mothers had diseases that could possibly have affected the growth pattern of their children during the neonatal period (eg diabetes mellitus) were excluded, as were subjects who were born preterm.

### Methods of measurement

The study team comprised 1 senior research assistant and 20 medical students who were precisely instructed in the method of taking weight, height, and HC measurements.

Methods of measurement were those recommended by Abdulrazzaq et al,^14^ and research assistants were trained to perform the measurements by the study authors. Weight was measured to the nearest 0.1 kg using a Detecto scale with a 140-kg capacity (Detecto Scales Inc., Brooklyn, NY, USA). Weight measurements were taken without shoes and in as little clothing as possible. To minimize errors in measurements, the weighing scales were checked before each session to ensure that the unloaded scale registered zero. The weighing scales were also frequently validated by weighing an object with known weight. Height was measured as the distance between 2 flat surfaces, using a stadiometer attached to the weighing scale. Standing height was measured to the nearest 0.1 cm. Each child stood erect and barefoot, with heels, buttocks, and back touching the stadiometer. Then, the horizontal indicator of the stadiometer was lowered until it firmly touched the crown of the head.^14^ HC was measured in children aged up to 5 years. The head was measured at the greatest circumference, which is slightly above the eyebrows and ear pinna and around the occipital prominence at the back of the skull. A disposable paper tape measure was used to give accurate data. The measurements were made 3 times to the nearest 0.1 cm in each individual, and the largest measurement was noted.

### Statistical analysis

We compared standards for normal (non-DS) children in the UAE and constructed growth charts for DS children in the UAE. For both purposes Cole’s LMS statistical method^15^ was used, which simultaneously allowed for development of smoothed curves and efficient calculation of z-scores. The LMS method is based on the use of Box-Cox transformations to normality^16^ through the calculation of a skewness parameter. The LMS parameters are the power in the Box-Cox transformation (L), the median (M), and the generalized coefficient of variation (S). Given these parameters and the assumption that the residuals follow a normal distribution, any desired percentile can be calculated. The following equations, where X is the value of the anthropometric variable and Z is the desired percentile in standard deviation units, give the value of X at the desired percentile:

$$X=M{\left(1+\mathrm{LSZ}\right)}^{\mathrm{1/L}};L\neq 0$$

Conversely, for any given value of X, the corresponding z score (Z) can be calculated as:

$$Z=[{\left(\mathrm{X/M}\right)}^{L}-1]/\mathrm{LS};L\neq 0$$

We used the above (standard) formula to calculate the z-scores for weight (height and HC were done analogously):

$$\begin{array}{ll} & \mathrm{Z-score}\\ & \;=[{\left(\mathrm{weight/M}\_\mathrm{weight}\right)}^{L\_\mathrm{weight}}-1]/[L\_\mathrm{weight}\times S\_\mathrm{weight}] \end{array}$$

Where L_weight, M_weight, S_weight are the age-specific anthropometric distribution parameter as derived from the UAE growth curves^3^ The method of maximum penalized likelihood generated values of L, M, and S parameters that were smoothed over age or height. These parameters were then used to construct the desired percentiles.

For ages 2 and over, the International Obesity Task Force (IOTF) cut-off points were used.^17^ LMS scores that were utilized to derive the graphs in our earlier paper^3^ were used to calculate z-scores for children with DS. Standard descriptive statistical and graphical methods, such as box plots, were used for analysis. For the construction of local LMS scores for UAE DS children, LMS Chartmaker (Harlow Healthcare, Tyne and Wear, United Kingdom) was used and, after visually inspecting several LMS combinations, the values of (4,6,4) were chosen, as these values demonstrated a good compromise between fit and smoothness. SPSS v.20 (IBM Corporation, Armonk, NY, USA) was used for all other data management and analysis.

## RESULTS

Given the lack of a DS registry when the study was conducted, the number of Emirati DS births in the population is not known. However, the number of Emirati live births in Tawam Hospital in Al Ain during the period of April 2008 to March 2014 was 21 712. In the same period, there were 58 Emirati live births with DS. Therefore, the incidence of DS in the Emirati population of Al Ain was determined to be 1 in 374 live births (26.71 Emiratis with DS in 10 000 Emirati live births). Chromosomal analyses were performed in 100 of the 182 patients studied. There were 95% trisomies, 2% Robertsonian translocations, 2% mosaics, and 1% 46,XX with features of DS. Table 1 describes these genetic traits in more detail.

**Table 1.** Result of chromosome analysis of 100 out of the 182 DS patients studied

Chromosome abnormality	Frequency
46,xx (features like down syndrome)	1
46,xy homogeneous trisomy 21 by Robertsonian 21-21 translocation	1
46,xy,der(21:21) (q10:q10), trisomy 21 by Robertsonian translocation between 2 chromosome 21.	1
47,xx,+21	38
47,xy,+21	57
Mosaic type trisomy 21 (33)/46,xy(17)	1
Mosiac 47,xx,+21 [10], 46,xx [25]	1

We collected data from 182 children and adolescents with DS, 113 boys and 69 girls, born in the period between 1994 and 2012. A total of 38 DS children were born between 1994 and 2000, and the rest (144) were born between 2000 and 2012. The numbers of subjects recruited for the study by area were 53 Emirati children with DS from Dubai, 25 from Abu Dhabi, and 104 from Al Ain (Table 2). The boy-to-girl ratio was 1.6:1, and ages ranged from 0 to 26 years. There were 1263 valid examinations of these 182 individuals with DS; 783 (63%) in boys and 480 (37%) in girls. Mean birth length was 47 cm in both sexes. Mean birth weight was 3.1 kg for boys and 2.9 kg for girls. Comparison of the lengths/heights of UAE DS children and those of UAE normal children from 0 to 13 years are shown in Table 3. Table 4 shows results of comparison of the weights of UAE DS children and those of UAE normal children from 0 to 13 years. In addition, Table 5 shows the HC of UAE DS children and standard UAE chart from 0 to 6 years. The IOTF cut-off points^17^ were used to examine prevalence of obesity and overweight in DS children from age 2 years to 16 years. The prevalence of overweight and obesity in subjects ≥10 years of age using these IOTF criteria were 32% and 19%, respectively. Table 6 shows the incidence of overweight and obesity in the study population using the IOTF cut-off points.

**Table 2.** Number of Emirati children with Down syndrome who attended the Health Centres, number fulfilling study entry criteria, number selected, and number entered into study in the 3 health centres in 3 cities of the United Arab Emirates

Centre	Number attending centre	Number fulfilling inclusion criteria	Number selected	Number did not attend	Number entered into study
Dubai	356	161	78	25	53
Abu Dhabi	154	72	30	5	25
Al Ain	229	206	150	46	104
Total	739	439	258	76	182

**Table 3.** Comparison between the mean lengths/heights of Down syndrome children and those of normal children from 0 to 13 years

	Girls			Boys		
	
Age, months	*n*	Length/Height, cm, Mean ±SD Down syndrome	Length/Height, cm, Mean Normal	*n*	Length/Height, cm, Mean ±SD Down syndrome	Length/Height, cm, Mean Normal
0–1.99	89	48.97 ± 3.8	52.5	149	49.81 ± 3.97	51.58
2–3.99	23	55.75 ± 4.56	56	61	56.18 ± 5.09	56.38
4–5.99	27	57.67 ± 5	59.52	47	62.08 ± 3.14	60.42
6–8.99	26	63.24 ± 3.61	63.43	46	64.93 ± 4.69	64.97
9–11.99	29	66.94 ± 3.46	67.92	38	70.81 ± 2.38	70.25
12–17.99	51	71.18 ± 5.1	74.32	91	73.73 ± 6.39	77.42
18–23.99	49	75.56 ± 5.61	81	83	78.45 ± 5.24	85.3
24–35.99	55	83.63 ± 3.99	93.53	75	84.96 ± 4.42	94.14
36–47.99	53	88.23 ± 5.02	100.31	58	90.60 ± 4.20	101.15
48–59.99	34	98.27 ± 4.02	106.25	62	96.79 ± 5.90	106.29
60–71.99	23	103.99 ± 5.88	111.32	52	103.41 ± 7.39	111.54
72–83.99	9	110.06 ± 5.03	116.69	37	109.42 ± 4.71	117.55
84–95.99	14	114.91 ± 4.97	123.18	38	112.54 ± 8.04	123.39
96–107.99	7	122.41 ± 3.03	126.7	11	118.52 ± 5.15	127.68
108–119.99	13	123.08 ± 6.29	131.32	18	122.18 ± 5.45	131.29
120–131.99	17	128.52 ± 7.33	137.45	14	130.16 ± 3.11	135.62
132–143.99	13	133.92 ± 3.11	142.37	9	136.02 ± 8.96	140.32
144–155.99	10	135.75 ± 5.58	148.28	5	136.88 ± 8.19	144.82

SD, standard deviation.

**Table 4.** Comparison between the mean weights of Down syndrome children and normal children from 0 to 13 years of age

	Girls			Boys		
	
Age, months	*n*	Weight, kg, Mean ±SD Down syndrome	Weight, kg, Mean Normal	*n*	Weight, kg, Mean ±SD Down syndrome	Weight, kg, Mean Normal
0–1.99	89	2.83 ± 0.71	3.56	149	3.11 ± 0.71	3.63
2–3.99	23	4.41 ± 0.95	5.01	61	4.49 ± 0.92	5.17
4–5.99	27	5.02 ± 1.21	6.01	47	6.1 ± 0.91	6.41
6–8.99	28	6.11 ± 1.27	7.28	46	6.95 ± 1.09	7.6
9–11.99	29	6.89 ± 1.43	8.55	38	8.44 ± 1.34	9.01
12–17.99	61	8.07 ± 1.2	10.13	91	8.61 ± 1.65	10.54
18–23.99	49	9.37 ± 1.68	11.61	83	9.62 ± 1.63	11.95
24–35.99	55	11.05 ± 1.32	13.32	75	11.45 ± 1.44	13.33
36–47.99	53	12.33 ± 1.42	14.72	58	12.97 ± 1.18	14.95
48–59.99	34	14.79 ± 2.30	16.2	62	15.64 ± 2.84	16.43
60–71.99	23	17.53 ± 3.33	17.8	52	17.30 ± 3.38	18.08
72–83.99	9	20.57 ± 1.53	19.53	37	19.16 ± 2.42	19.99
84–95.99	14	23.43 ± 3.96	22.05	38	20.77 ± 6.48	22.05
96–107.99	7	27.90 ± 4.20	23.96	11	23.49 ± 3.56	24.19
108–119.99	13	28.28 ± 6.55	27.12	18	27.88 ± 6.24	26.7
120–131.99	17	35.73 ± 9.22	31.64	14	34.19 ± 4.45	30.1
132–143.99	13	45.83 ± 5.89	35.29	9	39.22 ± 13.23	34.25
144–155.99	10	51.41 ± 12.91	40.23	5	40.04 ± 14.42	38.03

SD, standard deviation.

**Table 5.** Comparison between the mean Head Circumferences of Down syndrome children and those of normal children from 0 to 6 years of age

	Girls			Boys		
	
Age, Months	*n*	HC, cm, Mean ±SD Down syndrome	HC, cm, Mean Normal	*n*	HC, cm, Mean ±SD Down syndrome	HC, cm, Mean Normal
0–1.99	89	33.1 ± 2.04	34.82	149	33.98 ± 2.32	35.7
2–3.99	23	36.71 ± 1.31	30.55	61	37.38 ± 2.11	40.44
4–5.99	27	38.53 ± 1.69	41.27	47	39.48 ± 1.74	42.61
6–8.99	26	40.54 ± 1.49	43	46	41.6 ± 1.82	44.17
9–11.99	29	41.64 ± 2.02	44.33	38	43.59 ± 1.92	45.53
12–17.99	61	42.5 ± 1.72	45.58	94	43.88 + 1.97	46.78
18–23.99	49	44.37 ± 1.65	46.66	83	44.47 ± 1.88	47.84
24–35.99	55	45.76 ± 1.27	48.16	75	45.37 ± 1.51	49.01
36–47.99	53	45.98 ± 1.06	48.39	58	45.93 ± 1.23	49.64
48–59.99	34	46.10 ± 1.36	49.65	62	47.64 ± 1.72	50.47
60–71.99	23	46.20 ± 1.30	49.91	52	47.35 ± 1.85	50.76

HC, head circumference; SD, standard deviation.

**Table 6.** Prevalence of overweight and obesity in Down syndrome children from 2 to 16 years

	Normal		Overweight^a^		Obese^a^	
		
Age, years	*n*	% of total	*n*	% of total	*n*	% of total
2–2.99	123	94.6	6	4.6	1	0.8
3–3.99	100	90.1	9	8.1	2	1.8
4–4.99	82	83.7	12	12.2	4	4.1
5–5.99	59	78.7	11	14.7	5	6.7
6–6.99	39	83.0	8	17.0	0	0.0
7–7.99	42	80.8	8	15.4	2	3.8
8–8.99	13	72.2	3	16.7	2	11.1
9–9.99	18	56.2	12	37.5	2	6.2
10–10.99	15	48.4	10	32.3	6	19.4
11–11.99	6	27.3	6	27.3	10	45.5
12–12.99	4	26.7	3	20.0	8	53.3
13–13.99	2	40.0	2	40.0	1	20.0
14–14.99	1	7.7	1	7.7	11	84.6
15–15.99	1	14.3	2	28.6	4	57.1

^a^The International Obesity Task Force (IOTF) cut off points were used to define overweight and obesity.

**Figure 1.**
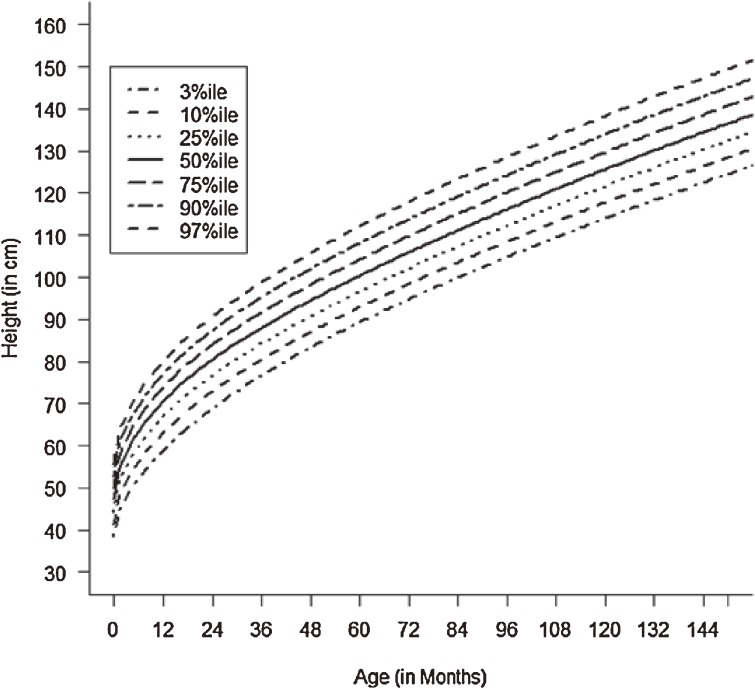
Line chart of height/length (cm) with age in UAE boys aged 0–13 years showing heights at the 3rd, 10th, 25th, 50th, 75th, 90th, and 97th centiles at different ages.

**Figure 2.**
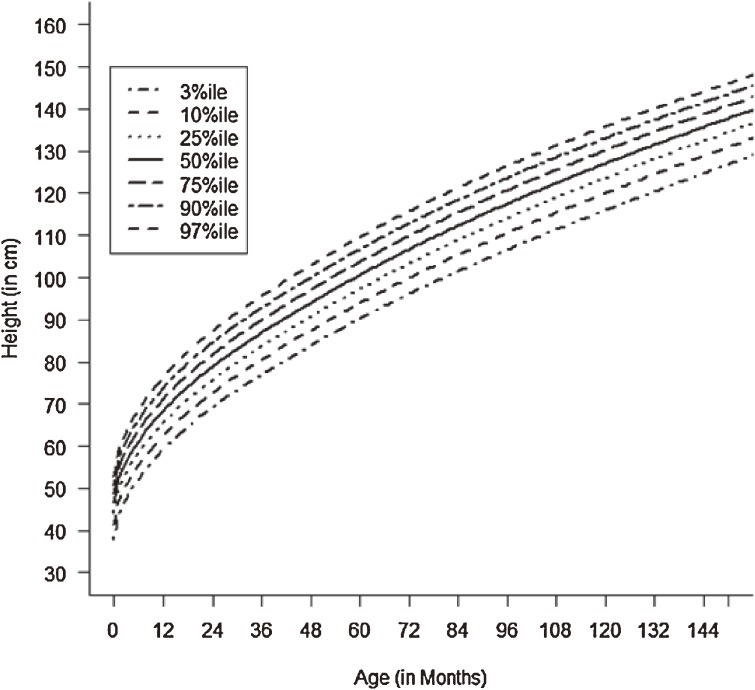
Line chart of height/length with age in UAE girls aged 0–13 years showing heights at the 3rd, 10th, 25th, 50th, 75th, 90th, and 97th centiles at different ages.

**Figure 3.**
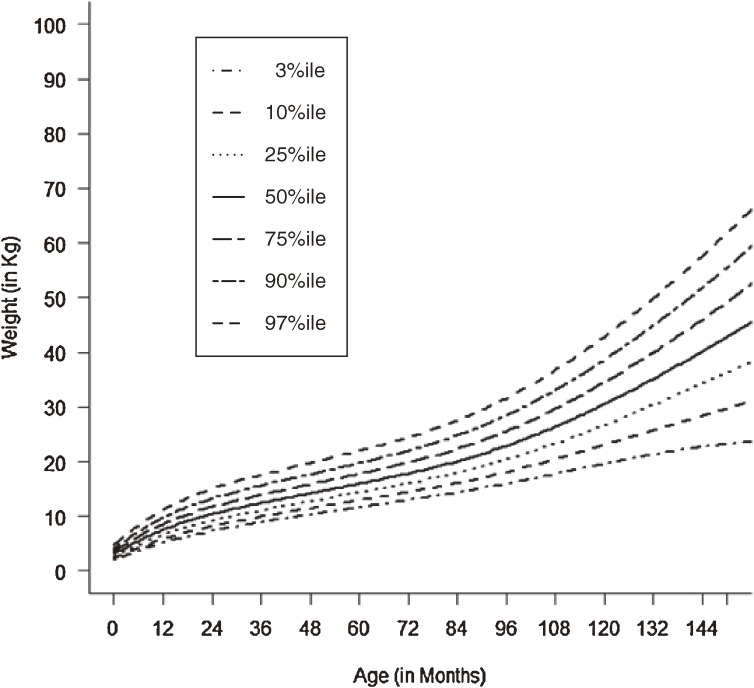
Line chart of weight (WT) with age in UAE boys aged 0–13 years showing weights at the 3rd, 10th, 25th, 50th, 75th, 90th, and 97th centiles at different ages.

**Figure 4.**
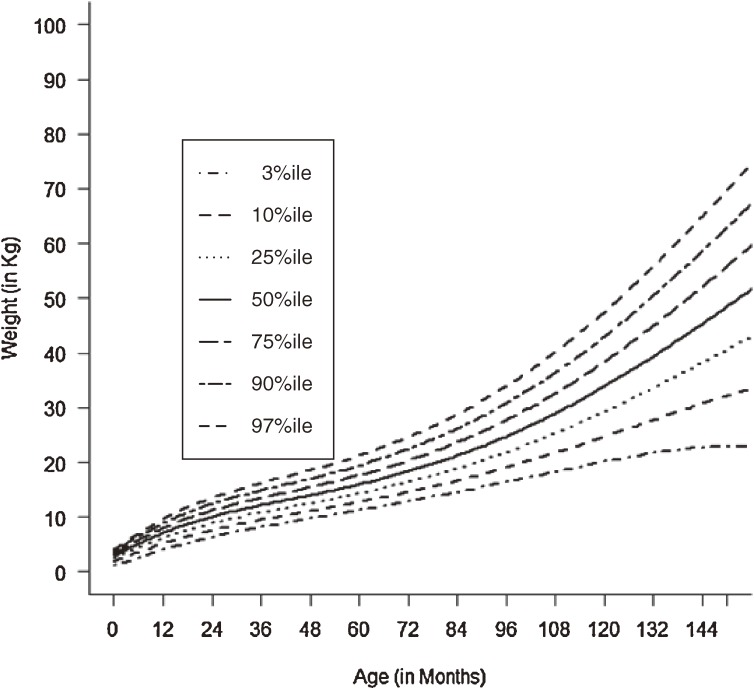
Line chart of weight with age in UAE girls aged 0–13 years showing weights at the 3rd, 10th, 25th, 50th, 75th, 90th, and 9th centiles at different ages.

**Figure 5.**
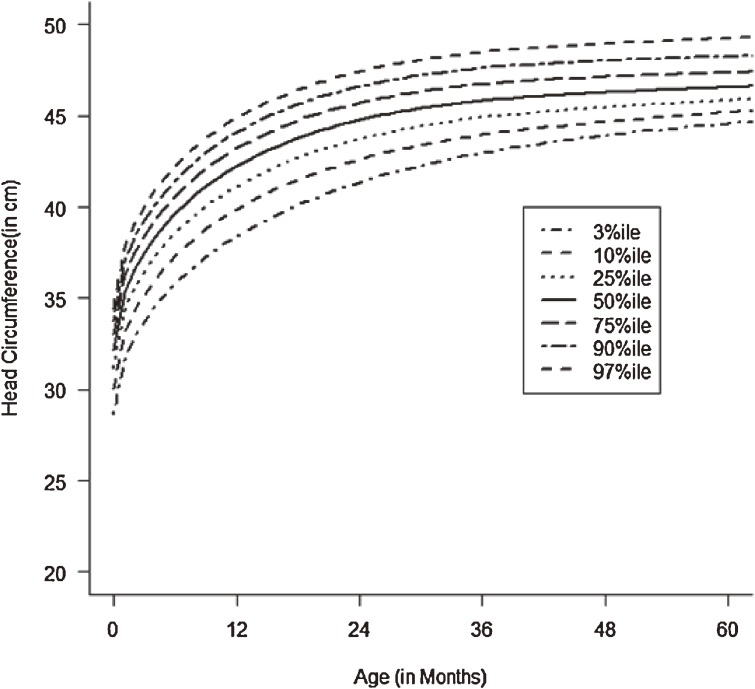
Line chart of head circumferences (HC) of UAE boys aged 0–60 months showing head circumferences at the 3rd, 10th, 25th, 50th, 75th, 90th, and 97th centiles at different ages.

**Figure 6.**
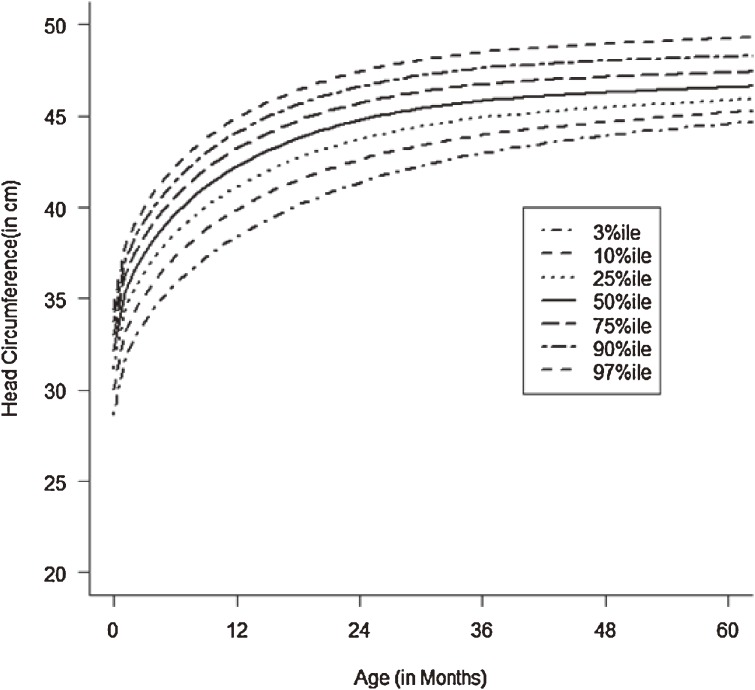
Line chart of head circumferences (HC) with age of UAE girls aged 0–60 months showing head circumferences at the 3rd, 10th, 25th, 50th, 75th, 90th, and 97th centiles at different ages.

**Figure 7.**
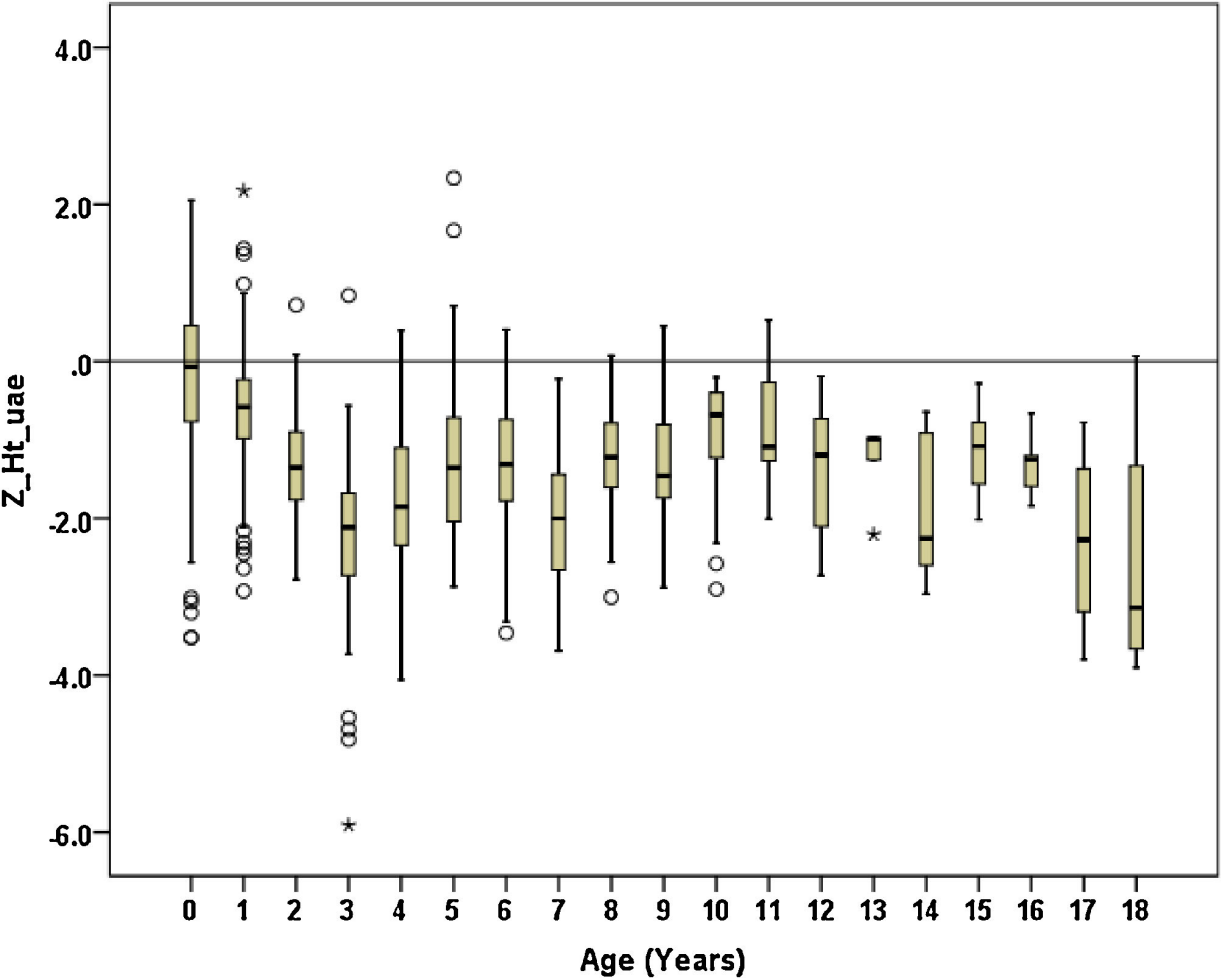
Demonstrates the boxplots of z-scores for different ages for height when compared with standard UAE population data.

**Figure 8.**
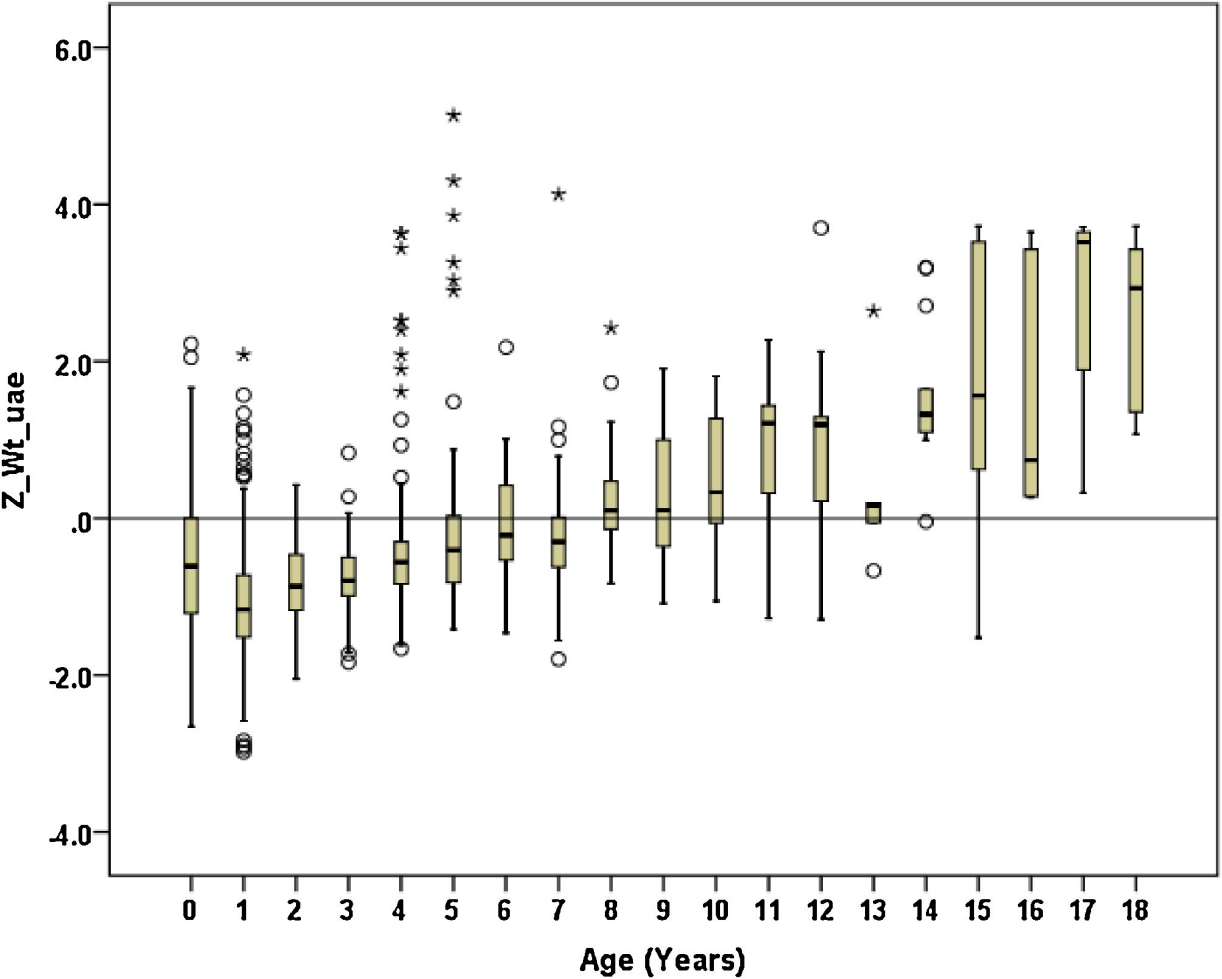
Displays the boxplots of z-scores for different ages for weight respectively when compared with standard UAE population data

Figures 1–4 show the weight-for-age and height/length-for-age reference percentiles for girls and boys aged 0 to 13 years. Figures 5 and 6 show the reference percentile charts for HC for age for girls and boys aged 0 to 72 months. Each figure shows the 3rd, 10th, 25th, 50th, 75th, 90th, and 97th percentiles, corresponding to SD scores of −1.88 to +1.88 after transformations to normality. Figures 7 and 8 are boxplots of z-scores for different ages for height and weight, respectively, when compared with standard UAE population data.^3^

## DISCUSSION

Growth charts exist for DS^8^^,^^10^^,^^18^^,^^19^ and for other chromosomal conditions like Turner syndrome,^20^ Noonan syndrome,^21^ and Prader-Willi syndrome,^22^ but none of these growth charts have been validated for the UAE. Growth is one of the best markers of health status both individually and for the population, and this is especially true among those with genetic disorders such as DS, given its associated organ dysfunctions. Although short stature is a characteristic feature of DS, the severity of this feature is influenced both by genetic factors and inherited parental factors. Other factors, such as other diseases affecting the child, may also influence growth. As in normal children, growth charts specific for children with DS are important tools in monitoring treatment efficacy and as routine medical follow up.

In order to create optimum country-specific growth charts, a longitudinal, prospective study based on repeated examinations of a large and representative group is needed. With a small population with low numbers of children with DS meeting entry criteria, it becomes almost impossible to gather sufficient numbers in each age group to create a valid growth chart. Therefore, in the present study, we used a mixed longitudinal (retrospective) and cross-sectional study in which we both collected data at different ages for each child, as in a longitudinal study, as well as conducted examinations of different children in the same age group, as in a cross-sectional study. This method is commonly used when growth is measured in specific groups with relatively few subjects.^8^^,^^19^^,^^23^^–^^25^

The present study has produced new charts of healthy growth of children with DS in the UAE. All children were selected nationwide from hospital-based outpatient clinics, providing standard care to children with DS. Selection was not biased, as all Emirati children with DS in the 3 cities in the UAE were invited for the study. The number of boys ultimately enrolled was higher than the number of girls, as more boys agreed to be studied; because of the small sample size, this sex differential could be a statistical shortcoming and not a characteristic of DS patients in the UAE. Since the number of children with DS in the UAE meeting the inclusion criteria is relatively small and would not be sufficient to construct a growth chart, we included all measurements of length/height, weight, and HC taken of all the children throughout their lifetime. Our new reference charts for UAE children with DS are representative of the population with DS. We had strict selection criteria for subjects with respect to their health. All children also received high-quality medical care, provided by the regional hospital-based outpatient clinics for children with DS.

Chromosomal studies on 100 of the 182 children studied revealed 95% of the children to be non-disjunction trisomy 21. The prevalence of this chromosomal abnormality is similar to that found in the England and Wales,^26^ Scotland,^27^ and France^28^ but higher than that of Belgium^29^ and Canada.^30^ Within the region, the prevalence of trisomy 21 is similar to that of Kuwait^31^ but lower than that of Qatar.^32^ Two percent of the chromosomal abnormalities were translocations, and 2% were mosaics. There was one patient with a normal karyotype (46,XX) of the peripheral blood leukocytes but had typical physical features of DS. This was likely a mosaic which may have showed up if skin fibroblast karyotyping would have been performed. In one study of DS in the UAE population,^2^ the prevalence was found to be 31.3/10 000 live births among Emiratis (1 in 374 Emirati live births), which is one of the highest prevalence rates of DS in the world, and the prevalence in the expatriate population was found to be 16.6/10 000 live births. In other Gulf countries, like Qatar, the prevalence is 19.5/10 000 live births,^32^ and in Saudi Arabia the prevalence is 18.6/10 000 live births.^33^ The sex ratio is skewed towards boys, with Qatar reporting a male:female ratio of 1.12:1 and the present study showing a ratio of 1.6:1. One of the reasons for this could be the small sample size, and another possible explanation being refusal of more girls to participate in the study.

Mean birth length in UAE children with DS was found to be slightly lower than the mean for the normal population, with z-scores slightly below zero. When the children with DS reached 3 years of age, the z-score was below −2.0, and this trend continued throughout the period during which measurements were made, with mean z-scores remaining below −2.0 in comparison with the normal population. Similarly, Turkish DS children were found to be −0.5 SD of the mean of the normal population at birth, but at 3 years of age, they were below −1.9 SD of the normal Turkish population and remained at that level throughout the measurement period.^24^

Growth velocity of children with DS has been known to decelerate during the period from 6 months to 3 years and at puberty.^8^^,^^13^ In the present study, we found that, like in Saudi Arabia,^33^ growth rate among children with DS differed significantly from that in normal children, and heights were significantly shorter than in the normal population at most ages. A similar trend was noted in the Netherlands^9^; in comparison with the general population growth standards, mean height of children with DS in the Netherlands was at −1.1 SD, decreasing to −2.2 SD at 3 years of age and remaining at this level until puberty, when the mean height achieved was at −2.9 SD.

As presented, the curves reflect an accurate picture of the Emirati DS population. However, we do not believe that they are a standard to be achieved, especially for weight in the older age groups, due to the high prevalence of overweight and obesity in that age group. Of note, there is a clear tendency toward being overweight in late childhood and the teenage years, a common finding in most other studies of DS children as well. The mean birth weight of our patients with DS was slightly lower than normal population (z-score −0.8). Final weight values were significantly higher than UAE standards for normal children. Z-scores increased continuously throughout childhood, becoming significantly higher than z-scores at lower ages at the age of 10 (z-score +1.2) and continuing to increase to the end of the period of measurement (18 years, z-score +3.0).

Rates of obesity and overweight were very high in children with DS, especially after age 10. Saudi data^34^ have shown that, in the first 2 years of life, children with DS were underweight, but by the age of 3 years, most tended to be overweight. However, the Saudi study only included children up to 5 years of age. In Sweden,^13^ the birth weight of DS male babies was at −1.2 SD and female babies was −1.5 SD when compared to normal Swedish neonates. However, at the age of 18 years, Swedish DS boys we at −0.4 SD and girls at −0.5 SD, which is very different from values in UAE DS children, who became more overweight with age, reaching a z-score of +3.0 at 18 years of age. The Dutch study did not include data on weight.^9^ The mean birth weights of Turkish^24^ DS children were −0.8 SD of their healthy counterparts, with mean weights at −1 SD, −0.7 SD, and −0.5 SD at 6 months, 3 years, and 5 years of age, respectively. The final mean weights were −0.3 SD (boys) and 0.5 SD (girls) for Turkish standards. These are similar to the Swedish data but again very different from the UAE data.

Although the lengths/heights of the DS infants and children were lower than those of the general Emirati population from birth through the rest of the ages, they only achieved significance after the age of one year. The growth pattern in DS infants and children showed impaired growth velocity, especially after age 1. In comparison with healthy Emirati children, mean length of DS children at birth was at −1.3 SD and at 13 years was −2.3 SD, indicating that children with DS show retarded growth even during pregnancy. Also, during the first year of life, they grow slower than the general population; the gap stays significant but relatively constant during the age interval of 1 to 13 years. This pattern has been shown to be the same in boys and girls with DS.

When we compared the heights of UAE DS boys with those of Swedish^13^ and Dutch^9^ boys with DS, we found that the Swedish and Dutch boys had similar growth to one another, but both were taller than UAE boys with DS at all ages, especially after the age of 5 years, and the final height achieved by UAE DS boys (mean height 158.46 ± 0.75 cm) was lower than both the Swedish (mean height 161.5 ± 6.2 cm) and Dutch (mean height 163 ± 6.2 cm) boys. Both Dutch and UAE girls were taller than Swedish girls, but, interestingly, Swedish and UAE girls were similar in their heights at all ages except in the first year of life. In comparison with Turkish children with DS, both Turkish girls and boys were longer than UAE DS children at birth, but heights then remained similar between Turkish and Emirati boys and girls up to 10 years of age. Turkish boys had faster growth at ages 12 to 16 years, but the final heights attained were lower in Turkish girls (mean height 144 ± 6.01 cm) than in the UAE DS girls (mean height 151.5 ± 2.12 cm), while the height attained by boys (mean height 160 ± 7.3 cm) was similar. It appears that Turkish boys had their pubertal growth spurt earlier than the UAE boys with DS. Similar trends were seen in girls, as Turkish girls were taller than UAE girls at 10 and 12 years of age, after which the UAE girls caught up and attained greater growth and higher final height.

The clinical conditions of DS, in which mental retardation is present, predispose children to being overweight.^35^ UAE children with DS are born weighing significantly less than normal UAE children and remain smaller until 8 years of age, but after 10 years of age, they become significantly heavier than the normal UAE population. While similar trends are seen in Swedish,^13^ American,^8^ and Turkish^25^ children with DS, overweight and obesity are significantly more prevalent and pose a greater problem in UAE DS children. Although heights in DS children were significantly shorter than in normal UAE children, weights were comparable and increased tremendously after 8 years of age. This means that UAE DS children put on relatively more weight than length or height from birth until adulthood, rendering them overweight and obese, especially after 8 years of age.

The mean HC of UAE children with DS was found to be smaller than that of healthy UAE children. When compared with DS children in other countries, both UAE girls (mean HC 32.5 ± 1 cm) and boys (mean HC 32.5 ± 1.5 cm) had smaller HC than their Dutch (mean HC 33.8 ± 0.04 cm in boys and 32.9 ± 0.03 cm in girls), Swedish (mean HC 33 ± 1.7 cm in boys and 32.5 ± 1.6 cm in girls), and Turkish (mean HC 34.2 ± 2.2 cm in boys and 34 ± 2 cm in girls) counterparts at birth. At later ages, HC measurements in UAE DS children were closer to those in Turkish DS children than to those in Dutch or Swedish children. Swedish and Dutch children also had higher HC measurements throughout the first 5 years of life.

The study has one major limitation that warrants mention. The total number of children with DS in our study is low; this corresponds to the number of UAE nationals, which is less than 1 million, even though the incidence of DS is 1/319 live births (31.3 per 10 000).

In conclusion we present for the first time centile charts for weight, height, and HC appropriate for children with DS in the UAE. Patterns of growth of DS children are biologically different from those for the general population. Early identification of a range of pathologies and prevention of overweight and obesity can be best achieved by using syndrome-specific charts. The charts presented here are informative and accurate. Differences throughout the age range have significant clinical implications. We recommend that the new charts be adopted as the standard reference for DS children in the UAE. The heights and HCs of UAE children with DS were smaller than values in the normal population. While weights of UAE children with DS at birth were also below the normal standard, after the age of 8, weights were significantly higher than normal. The pubertal growth spurt may be delayed in UAE children with DS. The final height achieved by UAE children with DS was shorter than that in Swedish and Dutch boys and girls with DS. The HCs in UAE children with DS were also less than HCs of Swedish and Dutch children with DS between 0 and 5 years.
